# Hysteroscopy: A necessary method for detecting uterine pathologies in post-menopausal women with abnormal uterine bleeding or increased endometrial thickness

**DOI:** 10.4274/tjod.66674

**Published:** 2016-12-15

**Authors:** Fatemeh Sarvi, Ashraf Alleyassin, Marzieh Aghahosseini, Marzieh Ghasemi, Sima Gity

**Affiliations:** 1 Tehran University of Medical Sciences Shariati Hospital, Clinic of Endocrinology and Infertility, Tehran, Iran; 2 Zahedan University of Medical Sciences, Pregnancy Health Research Center, Department of Obstetrics and Gynecology, Zahedan, Iran

**Keywords:** Abnormal uterine bleeding, endometrial thickness, post-menopause, hysteroscopy, endometrial biopsy

## Abstract

**Objective:**

To investigate the histologic and hysteroscopic findings of post-menopausal women with uterine bleeding and asymptomatic women with increased endometrial thickness equal or more than 5 mm.

**Materials and Methods:**

This cross-sectional study was performed between May 2014 and June 2015 on 110 post-menopausal women aged 40-82 years. The women were divided into two groups: Women with abnormal uterine bleeding (AUB group) and asymptomatic women with increased endometrial thickness (asymptomatic group).

**Results:**

Among the participants, 67 women had AUB and 43 women were asymptomatic. In the AUB group sensitivity, specificity, and positive and negative predictive values of hysteroscopy for normal findings were 98%, 100%, 100% and 90%, respectively. In the asymptomatic group, the same parameters were 98%, 100%, 100% and 85%, respectively. The sensitivity, specificity, and positive and negative predictive values of hysteroscopy for polyps and myomas were 100%. Also, the sensitivity, specificity, and positive and negative predictive values were 100% in hyperplasia cases found during hysteroscopy in both groups.

**Conclusion:**

Increased endometrial thickness in postmenopausal women with or without AUB is mostly due to benign lesions such as polyps and submucosal myomas. Hysteroscopy is a safe and reliable method for evaluating and treating these lesions.

## INTRODUCTION

Abnormal uterine bleeding (AUB) is as any kind of uterine bleeding in terms of duration, frequency, and volume. In postmenopausal women, women without a menstrual cycle for one year, any bleeding is abnormal. Postmenopausal bleeding has different causes including endometrial atrophy, polyps, myomas, endometrial hyperplasia, and endometrial carcinoma. Endometrial carcinoma is the most common malignancy of genital organs in women in developed countries. About 80% of endometrial cancers in post-menopausal women occur at ages of 50 to 65 years^(1)^. On the other hand 10% to 15% of women with post-menopausal bleeding have endometrial cancer^(2,3)^. Therefore, it is important to evaluate AUB in postmenopausal women very carefully. Measurement of endometrial line thickness by transvaginal sonography (TVS) is the first step to determine the need for further evaluations to rule out malignancy in these patients^(4)^. In case of endometrial thickness more than 4-5 mm in TVS of patients with postmenopausal bleeding, more evaluation is required to rule out cancer. Considering these values, the incidence of endometrial cancer with measurements thinner than this cut-off point is less than 1%^(5,6)^. There is no agreement on the described threshold of endometrial thickness to differentiate between normal and abnormal endometrial pathologies in postmenopausal women without bleeding^(7,8)^. Some guidelines and researchers have suggested that asymptomatic post-menopausal women with endometrial thickness of 4-5 mm or more do not need endometrial biopsy unless AUB occurs^(9,10)^. However, some researchers believe that postmenopausal endometrial thickness represents an increased risk of malignancy or other underlying pathologies, such as hyperplasia, polyps or myomas, and should be evaluated^(11)^. Hysteroscopy is a precise, easy, and quick method to assess and identify any intrauterine pathology with which we are able to observe the whole endometrial cavity and take adequate biopsies of any suspicious lesions. This procedure has recently been suggested as the best available method to evaluate the uterine cavity of women with endometrial line thickness with or without AUB^(12,13)^. Another advantage of hysteroscopy is the “see and treat” method in which simultaneous real-time macroscopic diagnosis of benign lesions and resection can be made^(1,14)^. This study was designed to investigate and compare the histologic and hysteroscopic findings of post-menopausal women with AUB and asymptomatic women with increased endometrial thickness.

## MATERIALS AND METHODS

This cross-sectional study was performed between May 2014 and June 2015 on post-menopausal women who were referred to a center in Tehran because of having endometrial thickness equal or more than 5 mm in TVS, with or without AUB. They were divided into two groups: women with AUB group and asymptomatic women with increased endometrial thickness (asymptomatic group). Menopause was defined as the absence of menstrual periods for more than 12 months. The study protocol was approved by our university’s ethics committee.

The inclusion criteria were: (1) being menopausal; (2) aged 40-82 years; (3) having uterine bleeding; and (4) having increased endometrial thickness (≥5 mm). The exclusion criteria were: (1) using hormonal replacement therapy, anticoagulants or selective estrogen receptor modulators; (2) having vaginal bleeding with a known cause in the vagina or cervix; (3) having any adnexal abnormality in TVS; (4) having any kind of cancer; and (5) being menopausal because of ovarian surgery. All participants signed an informed consent form before participating in this study. Transvaginal ultrasound was done for all participants. Endometrial line thickness was measured at the thickest part in the longitudinal plan of TVS with 7.5 MHz vaginal probe. The cut-off value of endometrial thickness was 5 mm or more. Adnexal regions also were assessed by TVS. If any mass or abnormality was observed in the adnexa, the woman was excluded from the study^(1,15)^. Of the 148 women who were referred to our center in the defined period, 110 women met the inclusion criteria. Among them, 67 women had AUB group and 47 women were asymptomatic with endometrial thickness (asymptomatic group).

Hysteroscopy was conducted in an outpatient setting with a 3.5-mm Storz hysteroscope and 30 degrees view by an operator with 8 years of experience in performing hysteroscopy. The media was normal saline and hysteroscopy was performed with or without complete or local anesthesia. The whole endometrial level and cavity were precisely and systematically evaluated using hysteroscopy. All findings were recorded accurately.

Hysteroscopic findings were defined precisely based on the specific findings detected during the procedure. Normal hysteroscopic findings included a normal, non-vascular smooth level. Abnormal findings included polyps, submucosal myomas, endometrial hyperplasia, and endometrial cancer^(16^).

Hyperplasic endometrium was defined as endometrium that was highly vascular, thick, and polypoid in appearance. Endometrial grooves became visible whenever it was pressed by the hysteroscope. Presence of abnormal vascular pattern and irregular fragile polypoid tissue with bleeding necrosis was defined as a sign of endometrial carcinoma^(17)^. Endometrial biopsy was performed for all participants with intrauterine lesions. Punch biopsies were conducted in women with atrophic endometrium who had no pathology in hysteroscopy. In women with pre-malignant or malignant lesions, targeted and random biopsies were performed. In women with polyps or myomas, the lesions were all resected using scissors or resectoscope, respectively. The biopsies were immediately placed in 10% formaldehyde and sent to the pathology laboratory. The pathologist knew nothing of the hysteroscopic findings. Histologic findings were defined as the final exact diagnosis standard of the endometrial pathology. The pathologic findings between the two groups and the percentages of each finding were analyzed. The hysteroscopy’s predictive value in endometrial lesions’ diagnosis was assessed based on the sensitivity, specificity, and positive and negative predictive values^(18,19)^.

### Statistical Analysis

Categorical and continuous variables are summarized as number (percentage) and mean, respectively. Hysteroscopy was considered as a screening test and endometrial biopsy as a standard. Data analysis was performed using the Statistical Package for Social Sciences (SPSS) version 20 (Chicago, IL, USA) by calculating sensitivity, specificity, and positive and negative predictive values.

## RESULTS

This study was conducted on post-menopausal women with a mean age of 57 years. Of the 110 participants with endometrial thickness equal or more than 5 mm, 67 (60.9%) had AUB. All 110 patients underwent hysteroscopy and endometrial biopsy. The hysteroscopic findings were categorized into five groups: normal, polyps, myomas, hyperplasia, and carcinoma (Table 1).

We compared the hysteroscopy and pathology results of all participants. Among 17 women who had normal hysteroscopy in both groups, one woman in each group had simple hyperplasia in histopathology and the other had atrophy (atrophy in our classification was part of normal results) (Table 2).

The most common finding on hysteroscopic evaluation was endometrial polyps in both groups (44.1% and 53.5% in AUB and asymptomatic groups, respectively). There were a total of 55 polyps and 20 myomas in both groups, which were confirmed by histopathology. Hyperplasia was found in 16 participants (11 and 5 in AUB and asymptomatic groups, respectively). This was confirmed with histology. Eleven cases were simple hyperplasia and five were complex or atypical hyperplasia. Three women in the AUB group and one in the asymptomatic group were suspected of having carcinoma in the hysteroscopy. Regarding the AUB group, the sensitivity, specificity, and positive and negative predictive values of the hysteroscopic view for finding normal results were 98%, 100%, 100% and 90%, respectively. In the asymptomatic group these parameters were 98%, 100%, 100% and 85%, respectively (Table 3). The sensitivity, specificity, and positive and negative predictive values of hysteroscopy for polyps and myomas were 100%. The sensitivity, specificity, and positive and negative predictive values were 100% for detecting hyperplasia with hysteroscopy in both groups. The sensitivity, specificity, and positive and negative predictive values of hysteroscopy for detecting carcinoma in the AUB group were 100%, 97%, 33% and 100%, respectively (Table 3). All lesions occupying the uterus (53 polyps and 20 uterine myomas) were diagnosed using hysteroscopy.

## DISCUSSION

The average of life expectancy for women has increased in recent years because of improved quality of life. Also, the number of women older than 60 years is increasing. In spite of the absence of vaginal bleeding, these women may still have uterine pathologies such as endometrial hyperplasia, polyps, uterine fibroids, adenomiosis or even endometrial cancer, some of which can be malignant. Up to now, there is no common agreement regarding the clinical management of increased endometrial line thickness in post-menopausal women.

In our study, the common cause of endometrial thickening and AUB was endometrial polyp, which is consistent with other studies^(1,20,21,22,23,24)^. Fortunately, polyps were not histologically malignant in our patients and this finding is in agreement with Loiacono et al.^(24)^ study. Elfayomy et al.^(2)^ showed that about 20% of polyps had malignant components hidden in their stem or center despite normal endometrial pathology in endometrial biopsy. Therefore, the authors suggested performing polypectomy via hysteroscopy in such women. On the other hand, 20 women of our study who only had increased endometrial thickness in TVS had submucosal myomas. Among them, 13 women had AUB and seven were asymptomatic. Therefore, we suggest that hysteroscopy be performed in all postmenopausal women with endometrial thickness ≥5 mm with or without AUB because of the successful resection of all polyps and sub-mucosal myomas without complications in these women^(1,17,24,25)^. It seems that more evaluations are needed in such cases because 86% of asymptomatic women with increased endometrial line thickness had underlying pathologic findings. This is in agreement with the studies of Loiacono et al.^(24)^ and Hartman et al.^(15)^.

In a study by Korkmazer et al.^(20)^ on post-menopausal women with increased endometrial thickness, all intra-uterine lesions including polyps and submucosal myomas were diagnosed only via hysteroscopy. Curettage was not able to detect all lesions in their study; 25 of 93 women with atrophic endometrium had endometrial polyp in hysteroscopy and direct biopsy. Also, Lee et al.^(25)^ compared biopsies obtained by curettage and hysteroscopy in post-menopausal women with bleeding. The authors concluded that performing curettage may not be reliable enough for evaluating endometrial pathology and suggested that endometrial biopsy with hysteroscopy must become the standard of diagnosis in these women. If endometrial biopsy is performed blindly, the detection of endometrial polyps or submucosal myomas might be missed. This leads to under diagnosis of this pathology during menopause. Therefore, the possibility of missing the underlying pathology will be eliminated by doing hysteroscopy^(20,26,27)^.

In our study, there was more endometrial hyperplasia in the AUB group than in the asymptomatic group (16% vs. 11.6%, respectively). Hysteroscopy in these patients enabled us to take targeted biopsies under direct vision. According to some studies, hysteroscopy did not have the desirable sensitivity compared with endometrial biopsy in women with endometrial hyperplasia. Thus, it was suggested to take endometrial biopsy under direct visualization during hysteroscopy^(2,28,29)^. The sensitivity, specificity, and positive and negative predictive values of hysteroscopy in diagnosing polyps, myomas, and endometrial hyperplasia were 100% in both groups. This finding is not in agreement with the diagnostic capability of hysteroscopy without biopsy in some studies^(2,30,31)^. Loiacono et al.^(24)^ diagnosed three women with endometrial carcinoma while studying women who had normal hysteroscopic findings. The sensitivity and positive predictive value of hysteroscopy decreased to 63% and 77% in their malignant cases. Our findings showed the same decrease in positive predictive value of hysteroscopy, which is consistent with their study. A limitation of our study was the small number of participants. Thus, the hysteroscopic values for endometrial malignancies’ diagnosis could not be assessed in the asymptomatic group. Of the women in AUB group, 1.5% had histologically confirmed endometrial cancer, and 5% had atypical or complex hyperplasia. However, the positive predictive value of hysteroscopy for diagnosing carcinoma was 35%. In some studies, the percentage of cancer in asymptomatic women with endometrial thickness more than 5 mm was 0.5-1.4%^(32,33,34,35)^.

In a study by Elfayomy et al.^(2^) endometrial carcinoma was not reliably detected with hysteroscopy. In their study, 7 of 14 women (16.9%) with endometrial cancer had suspicious findings in hysteroscopy, and no abnormality was found in the other half. According to the authors, the specificity of hysteroscopy without biopsy was low in diagnosing endometrial cancer. This finding has been reported in other studies too^(28,36^). Therefore, it is recommended to perform a biopsy even if hysteroscopy finds no abnormality to increase the validity of hysteroscopy in diagnosing endometrial hyperplasia and cancer in post-menopausal women with bleeding or with endometrial line thickness of 5 mm or more in TVS. In our study, we compared the results of hysteroscopy with the results of histopathology in post-menopausal women with AUB or endometrial thickness of 5 mm or more. According to our findings and other studies, endometrial thickness is often due to the presence of benign lesions such as polyps and submucosal myomas^(2,7,24)^. Our study showed that hysteroscopy is a safe and reliable method for evaluating benign endometrium lesions. In our study, all studied women had a histologic confirmation of their diagnosis, which makes our findings a desirable and optimal reference. Hysteroscopy is more accurate than transvaginal ultrasound or dilatation and curettage in the diagnosis of endometrial polyps and other space-occupying endometrial lesions in post-menopausal women^(20,37)^. Considering the failure rate of ultrasound or dilatation and curettage in detecting some endometrial lesions, evaluation of the endometrial cavity by direct visualization is critical in diagnosing space-occupying lesions in post-menopausal women.

## CONCLUSION

In contrast to some studies that state that doing hysteroscopy in asymptomatic post-menopausal women with increased endometrial thickness is not cost-efficient^(34,36,38)^ the present study showed that hysteroscopy is a safe and reliable procedure for evaluating benign lesions of endometrium such as polyps or submucosal myomas.

In order to rule out endometrial hyperplasia and cancer in postmenopausal women with bleeding or asymptomatic women with endometrial thickness, performing hysteroscopy and taking endometrial biopsies is recommended even if no lesion has been found. Further long-term prospective studies with more participants are necessary to find the optimum endometrial thickness in asymptomatic postmenopausal women.

## Figures and Tables

**Table 1:**
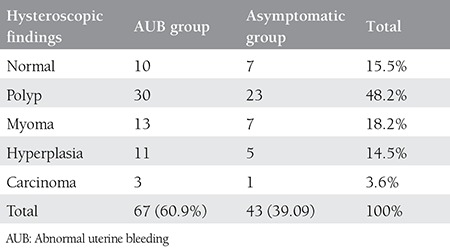
Hysteroscopic findings of our study groups

**Table 2:**
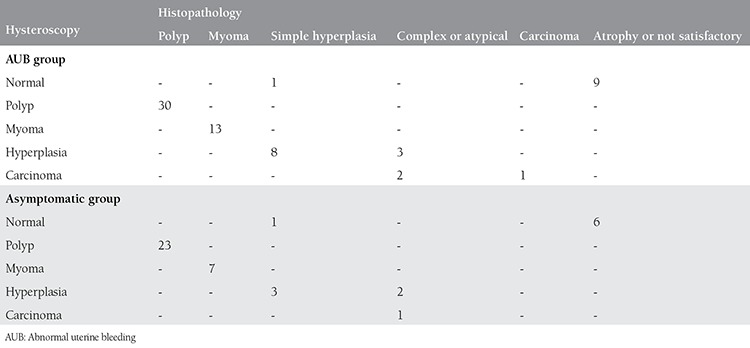
Comparison of the results of hysteroscopy and histopathologic findings of abnormal uterine bleeding and asymptomatic groups

**Table 3:**
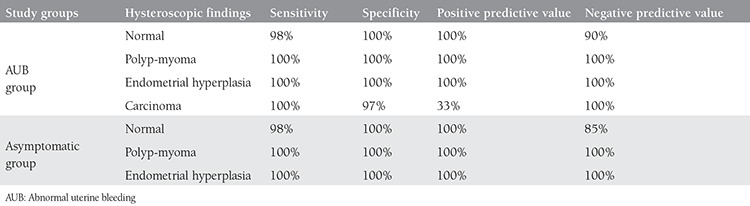
Sensitivity, specificity, and positive and negative predictive values of hysteroscopy
